# Forecasting air passenger traffic and market share using deep neural networks with multiple inputs and outputs

**DOI:** 10.3389/frai.2024.1429341

**Published:** 2024-10-10

**Authors:** Nahid Jafari, Martin Lewison

**Affiliations:** School of Business, Department of Management, SUNY–Farmingdale, Farmingdale, NY, United States

**Keywords:** air passenger demand forecasting, the U.S. Airlines Market, deep neural networks, multiple input-output, gated recurrent units

## Abstract

**Introduction:**

In this study, we address the challenge of accurate time series forecasting of air passenger demand using historical market demand data from the U.S. commercial aviation industry in the 21st century. Commercial aviation is a major contributor to the U.S. economy, directly or indirectly generating ~US$1.37 trillion annually, or 5% of annual GDP, and supporting more than 10 million jobs (Airlines for America, 2024). Over 1 billion passengers flew through U.S. airports in 2023 (Bureau of Transportation Statistics, 2024a). Using multiple correlated time series inputs predicts future values of multiple interrelated time series and leverages their mutual dependencies to enhance accuracy.

**Methods:**

In this study, we introduce a two-stage algorithm employing a deep neural network for correlated time series forecasting, addressing scenarios where multiple input variables are interrelated. This approach is designed to capture the influence that one time series can exert on another, thereby enhancing prediction accuracy by leveraging these interdependencies. In the first stage, we fit four Recurrent Neural Network (RNN) models to generate accurate univariate forecasts, each functioning as a single input-output model to predict aggregated market demand. The Gated Recurrent Unit (GRU) model was the top performer for our dataset overall. In the second stage, we apply the best fitted model (GRU Model) from Stage 1 to each individual competitor (disaggregated from the market) and then merge all input tensors using the Concatenate function.

**Results and discussion:**

We hope to contribute to the relevant body of knowledge with a deep neural network framework for forecasting market share among competitors in the U.S. commercial aviation industry, as no similar approach has been documented in the literature. Given the importance of the industry, there is potentially great value in applying sophisticated forecasting techniques to achieve accurate predictions of air passenger demand. Moreover, these techniques may have wider applications and can potentially be employed in other contexts.

## 1 Introduction

Demand for air travel is essentially driven by business and leisure activities. Economic downturns lead to a decrease in demand for both leisure and business travel, which in turn reduces the demand for air transport. Conversely, periods of economic growth increase the demand for leisure and business travel, which increases the demand for air transportation. According to the U.S. Department of Transportation's Bureau of Transportation Statistics (2024b), from 2000 to 2023, U.S. revenue passenger miles for domestic air travel increased by ~87%. The U.S. economy grew considerably over that same period.

Air transport demand forecasting is gaining increasing attention due to its significant economic impact and the inherent challenges of accurately predicting future demand. A reliable forecasting system is essential for effective airline decision-making, as it enables precise estimations of passenger demand. Given the complexity of air passenger demand—characterized by irregular patterns, high volatility, and seasonality—time series forecasting is an ideal approach. This method uses observed time series data from the past to predict future values over a specified look-ahead horizon, and is widely applied across fields such as finance, economics, and engineering.

To enhance forecasting accuracy, both univariate and multivariate time series methods are employed, especially when dealing with correlated time series—multiple interrelated time series that influence one another. Forecasting air traffic demand can be approached as a correlated time series problem, where multiple input variables are analyzed in relation to each other. This method recognizes that the value of one variable can affect and be affected by others, capturing the interconnected nature of the variables and resulting in more accurate forecasts. Despite its importance, there is a lack of extensive research on forecasting air traffic demand specifically as a correlated time series problem. Most existing studies have focused on simpler univariate models or have not fully explored the complexities introduced by multiple interrelated variables. Addressing this gap through more comprehensive studies that consider these correlations could significantly enhance forecasting accuracy and provide more robust tools for the airline industry.

Several factors add complexity to time series forecasting, including the sequence of input data, systematic patterns such as seasonality and stationarity, the length of the prediction horizon, and random noise. Among quantitative forecasting methods, econometric, statistical, and artificial intelligence approaches are particularly well-known for their application in these complex scenarios. Below, we briefly review these techniques in the relevant literature on air passenger demand forecasting.

### 1.1 Air passenger traffic forecasting approaches

There are many different methodological approaches to forecasting air passenger demand. Among quantitative forecasting methods, econometric, statistical, and artificial intelligence approaches are well-known. Below, we briefly review these techniques in the relevant literature on air passenger demand forecasting. For one, time series forecasting problems in commercial aviation can be examined as standard regression problems with time-varying parameters. Duval and Schiff (2011) and Abed et al. (2001) developed regression analysis models for forecasting the number of air passengers using various financial measures. Kim and Shin (2016) used basic regression analysis to consider causal relationships between air passenger demand and other variables. They analyzed big data from online search queries to determine which variables are reflected in short-term fluctuations of air passenger demand. Carmona-Benitez et al. (2017) proposed a forecasting approach using an Econometric Dynamic Model (EDM) to estimate passenger demand in the Mexican air transport industry. They applied the panel data Arellano-Bover method to calibrate the EDM, which was validated by the Sargan test and the Arellano-Bond Autocorrelation test. Hsiao and Hansen (2011) modeled city-pair air passenger demand at the route level using a type of Discrete Choice Method (demand assignment). Discrete Choice Methods are widely used for the analysis of individual choice behavior. Holt-Winters and AutoRegressive Integrated Moving Average (ARIMA) models are two practical methods among statistical time series forecasting models. The Holt-Winters method (Holt, 2004), which uses exponential smoothing, is an effective approach to forecasting seasonal time series. Bermudez et al. (2007) forecasted the number of air passengers in the UK using monthly air passenger data based on a smoothing method. Grubb and Mason (2001) presented a modified version of the Holt Winters method and Dantas et al. (2017) combined Holt Winters with Bootstrap aggregating (also called Bagging, a well-known machine learning technique) to improve the accuracy of air passenger forecasts. The ARIMA model (Box and Pierce, 1970) is one of the most prominent among univariate time series forecasting models which include other models like autoregression (AR), moving average (MA), and Autoregressive Moving Average (ARMA), another model which adapts various exponential smoothing techniques (McKenzie, 1984). ARIMA models are rarely used in high dimensional multivariate time series forecasting due to their high computational cost, but Tsui et al. (2014) employed the Box–Jenkins ARIMA methodology for forecasting Hong Kong's passenger demand (using data between 1993 and 2011) and projected the demand's future growth trend for the period of 2011–2015. In addition, Vector AutoRegression (VAR) is used for multivariate time series, and Support Vector Regression (SVR) is a linear model for univariate time series forecasting (Cao and Tay, 2003).

#### 1.1.1 Artificial intelligence approaches

Artificial intelligence (AI) techniques have also been investigated in air transportation forecasting. AI is a broad field that encompasses the development of systems or machines that can perform tasks that typically require human intelligence. These tasks include learning, reasoning, problem-solving, understanding natural language, and perception. AI includes a wide range of techniques such as rule-based systems, decision trees, genetic algorithms, machine learning, and neural networks, and it can be applied in a wide variety of complex computational domains. Neural networks in AI are capable of learning patterns and revealing the tendencies of the series. Brazilian air transport demand forecasting using neural networks has been studied by Alekseev et al. (2002) and Alekseev and Seixas (2009). The studies found that neural processing outperforms the traditional econometric approaches. Srisaeng et al. (2015) developed and empirically examined genetic algorithm optimization models (an alternative artificial intelligence-based approach) for forecasting Australia's quarterly domestic airline passenger demand. To predict short-term air passenger traffic, Xiao et al. (2014) combined singular spectrum analysis for identifying and extracting the trend and seasonality of air transport demand with an adaptive-network-based fuzzy inference system (another artificial intelligence technology) to deal with the irregularity and volatility of demand. Papageorgiou and Poczeta (2017) proposed a two-stage model for multivariate time series prediction based on the efficient capabilities of evolutionary fuzzy cognitive maps (FCMs) enhanced by structure optimization algorithms and artificial neural networks (ANNs). In the first stage of the model, an evolutionary FCM is constructed automatically from historical time series data using a genetic structure optimization algorithm, while in the second stage, the produced FCM defines the inputs in an ANN, which next is trained by the back propagation method with momentum and the Levenberg-Marquardt algorithm on the basis of available data. Chai and Lim (2016) presented a forecasting model of cyclical fluctuations of the economy based on the time delay coordinates embedding method. The model uses a neuro-fuzzy network with weighted fuzzy membership functions. Martinez et al. (2018) proposed a new strategy that forecasts every different season using a different specialized k-nearest neighbors (kNN) learner. Each kNN learner is specialized because its training set only contains examples whose targets belong to the season that the kNN learner is able to forecast. Wang and Han (2015) presented an improved extreme learning machine featuring a simple structure and good performance for the online sequential prediction of multivariate time series. Carson et al. (2011) found that in air travel demand forecasting, airport-specific forecasts perform better than forecasts using aggregate data. Zhang and Qi (2005) studied the effectiveness of data preprocessing, including deseasonalizing and detrending, on neural network modeling and forecasting performance. They found that neural networks are not able to capture seasonal or trend variations effectively with the unpreprocessed raw data and either detrending or deseasonalizing can dramatically reduce forecasting errors. We now discuss the approach used in our analysis, deep learning.

### 1.2 Deep learning: an overview

Deep learning, which has received an increasing amount of attention in time series analysis, is a branch of machine learning, which is itself a subset of AI. While AI is the overarching field concerned with creating intelligent systems, DL is a specific approach within AI that uses complex neural networks to process and learn from data. It focuses specifically on neural networks with many layers (hence the expression “deep”) and is used for learning from large amounts of data. DL encompasses models and architectures that learn optimal features from data by capturing increasingly complex representations of the data with combinations of layers of nonlinear data transformations (LeCun et al., 2015; Goodfellow et al., 2016). The two major architectures of deep neural networks involved in DL are Convolution Neural Networks (CNNs), which are appropriate for spatial data, object recognition, and image modeling, due to their ability to detect local patterns in data. Recurrent Neural Networks (RNNs)are suitable for sequence modeling. RNNs significantly enhance the capabilities of the feed-forward network with recurrent memory loops, which take the input from the previous and/or same layers or states RNNs are designed to handle sequence data, making them suitable for tasks involving time series and language modeling due to their capability to maintain temporal dependencies in the data through their feedback loops and internal states (Michelucci, 2018).

Time series forecasting (as Supervised Learning) using deep neural networks has been studied since early work was done using naive RNNs (Connor et al., 1991) and hybrid models (Zhang et al., 1998; Zhang, 2003). Models have progressed from Zhang (2003) combining ARIMA and Multilayer Perceptions (MLPs), to the recent combination of vanilla RNN and Dynamic Boltzmann Machines in time series forecasting (Dasgupta and Osogami, 2017). Yu et al. (2017) also proposed a deep learning approach to forecast short-term and long-term traffic patterns. They applied a deep neural network based on LSTM to forecast peak-hour traffic and managed to identify unique characteristics of the traffic data. They further improved the model for post-accident forecasting with a Mixture Deep LSTM model. Lai et al. (2017) also proposed a deep learning framework designed for multivariate time series forecasting, namely Long– and Short–term Time-series Network (LSTNet). LSTNet uses CNNs and RNNs to extract short-term local dependency patterns among variables and to discover long-term patterns for time series trends. In general, deep learning methods work better if we deal with huge datasets and if sufficient training data is available. By comparison, statistical forecasting methods (such as ARIMA/SARIMA and Holt-Winters) are computationally expensive, are practical only for small datasets, and are useful only in cases of univariate prediction.

In this paper, we propose a deep neural network framework for forecasting correlated time series data for the problem of predicting market share among competitors in an industry. We will deploy the proposed method using data from the U.S. commercial aviation industry. We examine various deep learning models to predict air passenger demand for U.S. domestic airlines as single and multiple inputs and outputs forecasting. We implement the models with Keras, the Python deep learning library. No previously reported study appears to address this problem. Our contribution to this literature is to develop a more accurate deep learning model upon which to ground univariate and multivariate forms of this forecasting problem. In Section 2, we first describe the data and then discuss preparations made to the data. In Section 3, we propose the framework of the deep learning models of our data and then identify the best fit. Finally, in Section 4, we summarize the model's performance and discuss its strengths and drawbacks in Section 5.

## 2 The data

### 2.1 Data description and analysis

In this study, we work with air passenger demand data from the U.S. domestic airlines over the period 2001–2023. Table 1 shows the source of the data and descriptions of key characteristics. We obtained the data from the U.S. Department of Transportation Bureau of Transportation Statistics (Bureau of Transportation Statistics, 2024), which provides comprehensive data on airlines in the U.S. domestic and international markets.

**Table 1:** Source of the study data and detailed description of key factors, along with descriptive statistics for U.S. airline passenger demand, 2001–2023.

**The three U.S. domestic carriers categories**			
The U.S. domestic airlines are categorized into three classes of carriers: Legacy, Low Cost Carriers, and Other, based on common characteristics and history. Legacy airlines began operations before 1978, at a time when fares, routes and market entry were federally controlled. The 1978 Airline Deregulation Act lifted a lot of restrictions for new airlines to enter the market and led, to a major expansion of the industry. All Low Cost Carriers entered the market under the new regime, when the circumstances allowed for low-cost no-frills strategies to be adopted. The LCCs focus on charging passengers only for minimal services and have consistently targeted passengers on short trips who require only small, hand-carried luggage.			
**The airlines abbreviation**			
Legacy	American (AA), Continental (CO), Delta (DL), Northwest (NW), US Airways (US), United (UA), America West (AW).		
Low Cost Carrier (LCC)	Southwest (WN), jetBlue (B6), AirTran (FL), Frontier (F9), Virgin America (VX).		
Other	Alaska (AS), Hawaiian (HA), Spirit (NK), Allegiant (G4).		
Note: American West merged with US airways in 2006: US Airways merged with American Airlines in 2015; Continental merged with United in 2012; Northwest merged with Delta in 2010; AirTran merged with			
Southwest in 2015.			
**Sources**			
Description		Type	Reference
Number of passengers		Monthly	Air Carriers : T-100 Domestic Segment (U.S. Carriers) Bureau of Transportation Statistics (Bureau of Transportation Statistics, 2024).
**Descriptive statistics**			
Monthly number of passengers, 2001–2023			
Measures	Values	Measures	Values
Mean	55,639,607	Range	73,407,995
Median	56,583,775	Minimum	2,924,771 (April 2020)
Standard deviation	10,533,808	Maximum	76332766 (July 2023)

**Figure 1:**
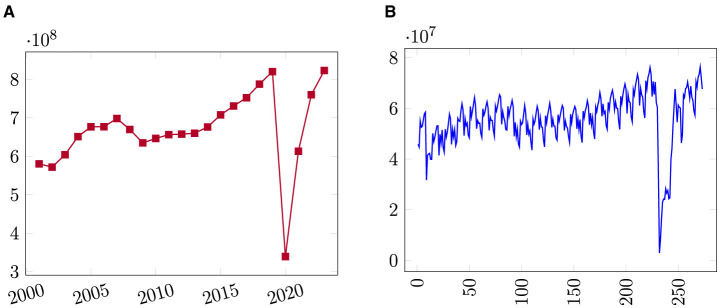
Line plot of U.S. domestic airline passenger demand from 2001 to 2023. **(A)** Annual passengers, **(B)** Monthly passengers.

Figure 1 offers a simple graphic depiction of the data. Annual passenger demand is plotted in Figure 1A and monthly passenger demand is plotted in Figure 1B. In the plot of annual passenger demand, only four years of declines in passenger totals are apparent, corresponding to reductions in the wake of the terror attacks of September 11, 2001 (2002), during the Great Recession (2008–2009), and the collapse of air travel in the first full year of the COVID-19 pandemic crisis (2020).

The monthly air passenger demand data from U.S. domestic airlines passenger traffic between January 2001 and September 2023 is plotted in Figure 1B. It shows that the demand for air travel is seasonal and cyclical. Demand spikes, as expected, in the summer months of June, July, and August. The dataset contains an aggregated number of air passengers for each month with no breakout of carrier (airlines). The dataset feeds single input-output models that we describe in Section 3. We will consider passengers carried by the currently operating U.S. domestic airlines every month when we predict air passenger traffic for individual routes between specific airport pairs to feed a multiple input-output model that we describe in Section 3.

Jafari (2022) attempted predictive analysis of U.S. domestic air passenger demand data from January 2001 to April 2021, but volatility resulting from the COVID-19 pandemic caused interruptions in the forecasts and produced inadequate results. Therefore, here we include the data only from 2001 to 2019 to feed our models. Bypassing the irregular data years is warranted because the aim of this work is developing a general model for correlated time series forecasts.

## 3 Methodology

This study forecasts air traffic in a market using multiple time series inputs (variables) while considering their correlation with each other, a method known as correlated time series forecasting. A fundamental characteristic of this approach is recognizing that the values of a time series variables can be influenced by the values of other time series variables. To enhance prediction accuracy, we are designing a deep neural network framework tailored to capture these interdependencies among the variables. The problem of correlated time series forecasts that aim to estimate multiple values (outputs) simultaneously given multiple inputs has been studied by Cirstea et al. (2018). Fox et al. (2018) has also investigated multi-output deep architectures for multi-step forecasting.

The general framework of our proposed deep neural network model for correlated time series forecasts is presented in Algorithm 1. The algorithm develops in two stages. In Stage 1, we fit an accurate univariate time series forecast model as a single input-output model to predict aggregated demand for a market. In Stage 2, we use the fitted model from Stage 1 for each competitor (disaggregated from the market), and then merge (combine) all of the input tensors using the Concatenate function. Afterward, for each competitor, a hidden layer makes connections between models.

**Algorithm 1:** Two stages Deep Neural Network Forecast model.

**Stage 1: Single Input-Output Forecasting**
Fit a model for univariate (aggregated) data
• ◇ Input layer (univariate data)
• ◇ Hidden Layer 1 (No. Neurons, Activation
Function, No. Epochs, No. Batches, Optimizer)
• ◇ ⋯
• ◇ Hidden Layer n (No. Neurons, Activation
Function, No. Epochs, No. Batches, Optimizer)
• ◇ Output Layer (1 neuron)
**Stage 2: Multiple Input-Output Forecasting**
For each competitor *i*
Model-*C*_*i*_=Run the fitted model in *Stage*1
Merge all Model-*C*_*i*_ using Concatenate
Hidden Layer 1
...
Hidden Layer *i*
Output Layer (1 neuron)

**Figure 2:**
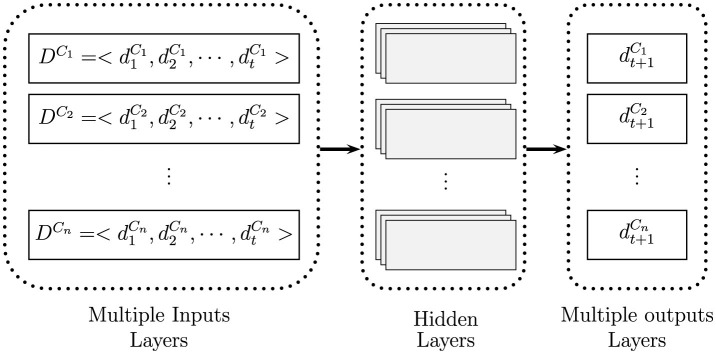
Feeding process of a correlated time series forecast as a multiple imputes, deploying hidden layers, and then generating multiple outputs (knowing $D^{C_{i}}$ is demand for competitor *i* in a market).

To elaborate the complex network for the multiple input-output forecast model expressed in Stage 2, we show the associated neural network architecture in Figure 2. Suppose we have *n* correlated time series represent demand (or sale) for a product (or a service) in a market by *n* competitors of a market over time as $D^{C_{i}}=<d_{1}^{C_{i}},d_{2}^{C_{i}},\cdots \;,d_{t}^{C_{i}}>$ where *i* = 1, ⋯ , *n*. If the demand for one competitor's offerings increases, the demand faced by all other competitors decreases. As we explained above, we are interested in forecasting individual demand for each competitor in the future simultaneously ($d_{t+1}^{C_{i}}$). Multiple input-output forecasting involves predicting multiple output variables using multiple input variables.

To apply the method, we use the U.S. air passenger traffic dataset previously introduced. Initially, we construct an accurate univariate forecast model focusing solely on monthly passenger demand as the single input-output model (shown in Figure 1B). We will then extend the developed model into a multiple input-output variables to predict monthly passenger demand for various competitor airlines for an individual route.

### 3.1 Single input-output forecasting model

A mathematical notation for a univariate time series forecasting model can be represented as follows:


$${Ŷ}_{t+h|t}=f\left(Y_{t},Y_{t-1},...,Y_{t-k};\theta \right)$$

where

Ŷ_*t* + *h*|*t*_ represents the forecast of the variable *Y* at time *t* + *h* given information up to time *t*.

*Y*_*t*_, *Y*_*t*−1_, ..., *Y*_*t*−*k*_ denote the past values of the time series up to lag *k*, where *k* represents the number of lagged observations considered in the model.

Function *f*(.) is the forecasting function that maps past observations to the forecasted value. The function is fitted by learning from the training data, which may involve multiple layers and activation functions.

Theta (θ) represents the parameters of the forecasting model, which are estimated based on the historical data.

Prior to specifying the forecasting function *f*(.), a recurrent neural network (RNN), we prepare our univariate time series dataset, the monthly number of passengers over the specified period, 2001-2019. We begin by applying a lag difference to our data to remove seasonal effects. The lag represents the number of periods after which the seasonal cycle repeats. In this case, the lag of our time series dataset is 12, corresponding to the monthly frequency of the data. Therefore, the difference variable is set to 12, which subtracts the value at each time step from the value 12 steps earlier, effectively removing seasonal patterns from the data. The lag difference operates by subtracting the value of period *t* − 12 from the value of period *t* in the series.

Next, we reframe our data from a time series to a supervised learning dataset. Supervised learning is where an algorithm is used to learn the mapping function from the input variables (*x*) to the output variable (*y*). The goal is to approximate the true underlying mapping so well that when you have new input data (*x*), you can accurately predict the output variables (*y*) for that data.

To design a best–fitting deep learning model using Keras on our datasets, we divide the data into three sets for training, validation, and test. To avoid overfitting by reusing the test set over and over again during the model selection, we partition our data of 228 observations to a training set (11 years), a validation set (4 years) and a test set (4 years). Once we find satisfactory hyper-parameter values, we retain the model from the completed training set and obtain a final performance estimate using the independent test set.

Table 2 presents a comparison of the four recurrent neural network (RNN) models developed for the univariate forecasting problem. Model 1 includes an LSTM layer with a SoftPlus activation function, followed by a dense layer with a ReLU activation function. The dense layer serves as the single output layer for making one-step predictions. The model is configured with 50 neurons, trained over 400 epochs with a batch size of 100, and optimized using the Adam optimizer.

**Table 2:** The details of our four different forecasting models.

**Model name**	**Input layers**	**Activation function**	**No. neurons**	**No. epochs**	**No. batches**	**Optimizer name**	**Loss**	
							**RMSE**	**MAPE (%)**
1	LSTM	Softplus	50	400	100	Adam	1,557,364	2.203
	Dense	Relu						
2	Conv1D	Relu	100	200	100	Adam	4,044,128	5.686
	Conv1D	Relu						
	MaxPooling1D	—						
	Flatten	—						
	LSTM	Relu						
	Dense	Relu						
3	Convlstm2D	Softplus	200	100	100	Adam	3,547,920	4.898
	Flatten	—						
	Dense	Relu						
4	GRU	Softplus	50	200	100	Adam	1,299,472	1.806
	Dense	Relu						

The last two columns present the average loss (forecast error) of the models in 100 repeats. Note that we use Dense(1) as the output layer in each models.

Note that the Rectified Linear Unit (ReLU) activation function is defined as: *ReLU*(*x*) = max(0, *x*). In simpler terms, the ReLU function outputs the input directly if it is positive; otherwise, it outputs zero. The SoftPlus activation function is defined as *softplus*(*x*) = ln(1 + exp(*x*)) in which a smooth approximation of the ReLU activation function. The output of the SoftPlus function is always positive, and it increases monotonically as the input increases. The Adam optimizer, short for Adaptive Moment Estimation, is a version of stochastic gradient descent and an advanced optimization algorithm designed for training deep learning models.

Model 2 integrates Convolutional Neural Networks (CNN) with Long Short-Term Memory (LSTM) networks. It comprises two convolutional layers, followed by a max pooling layer, a flattening layer, an LSTM layer, and a dense layer, all utilizing the ReLU activation function. The model is configured with 100 neurons, trained over 200 epochs with a batch size of 100, and optimized using the Adam optimizer.

Model 3 features a ConvLSTM layer, a specialized variant of LSTM that employs the SoftPlus activation function. Additionally, the model includes a flattening layer and a dense layer in its hidden structure utilizing the ReLU activation function. Configured with 200 neurons, the model is trained over 100 epochs with a batch size of 100 and optimized using the Adam optimizer.

Our final model, Model 4, is an RNN-based architecture that includes a GRU layer with the SoftPlus activation function, followed by a dense layer utilizing the ReLU activation function. The model is configured with 50 neurons, trained over 200 epochs with a batch size of 100, and optimized using the Adam optimizer.

We examine the accuracy of the models by computing two measures of the forecast error, the Root Mean Square Error ($RMSE_{test}=\sqrt{1/m\underset{i}{\sum }{\left({ŷ}^{\left(test\right)}-y^{\left(test\right)}\right)}_{i}^{2}}$ where ŷ^(*test*)^ gives the prediction of the model on the test set), and the Mean Absolute Percentage Error ($MAPE_{test}=1/m\underset{i}{\sum }|\frac{{ŷ}^{\left(test\right)}-y^{\left(test\right)}}{y^{\left(test\right)}}{|}_{i}\times 100\%$). The two error measures show us the variance between the estimated forecast of the models and the actual observed data. Note that the RMSE is useful when we compare the performance of different forecast models, while the MAPE expresses the forecast variance as a percentage of the actual. The error measures presented in Table 2 are the average of 100 repeated forecasts. Table 2 shows that Model 4 has the lowest loss among all of the tested models. Model 1, the Long Short Term Memory (LSTM) model, has the second lowest loss. Thus the two RNN models outperform the other deep network models for our dataset. The MAPE of Model 4 shows that the forecast is off by 1.806% on average.

Based upon the results obtained from our experiments in fitting a model, we select Model 4 as the fitted model to predict air passenger traffic. Having the satisfactory hyper-parameter values, we retain the best–performing model from the completed training set (2001–2013) and run a final performance estimate using the test set (2014–2019). The Root Mean Square Error (RMSE) is equal to 1, 175, 675 and the Mean Absolute Percentage Error (MAPE) is equal to 1.640%. To examine the performance of Model 4, we deploy the two popular statistical (classical) forecasting models, SARIMA and Holt-Winters on our data and we obtain a forecast loss for SARIMA (ARIMA (1, 0, 1) × (1, 1, 1)_12_) of *RMSE* = 1, 737, 155 and *MAPE* = 2.143%, and for Holt-Winters *RMSE* = 1, 696, 176 and *MAPE* = 2.218%. The comparison makes clear that the developed GRU model outperforms the SARIMA and Holt-Winters models. We now progress to the Multiple Input-Output model.

### 3.2 Multiple input-output forecasting model

We extend the single input-output (univariate) forecast model to develop a multiple input-output forecast model for predicting air traffic on specific routes between airport pairs (from an origin airport to a destination airport) for various airlines operating on those routes concurrently. Since passenger demand on a route for any one airline can influence the number of passengers carried by other airlines, the passenger traffic across different airlines is interrelated. To capture these dependencies effectively, we introduce a new variable that represents the total number of passengers across all airlines. By summing up the demand across all airlines, this new variable provides a comprehensive measure of overall passenger traffic. Incorporating this aggregate measure into the model allows it to learn the relationships and interactions between the individual inputs (demand for each airline) more effectively. This approach uses a first-degree polynomial approximation, which simplifies the modeling of these relationships by focusing on linear dependencies. As a result, the model can better understand how variations in the total passenger count influence and relate to the demand for each specific airline, thereby improving the accuracy and relevance of its forecasts. To handle this problem, a model with multiple inputs and outputs, we use the Keras functional Application Program Interface (API) vs. the Keras Sequential. The associated neural network for the explained problem would be designed.

**Figure 3:**
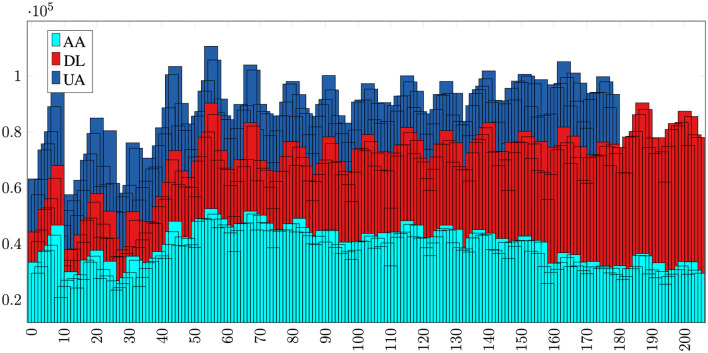
The market share and passenger demand correlation among three competing airlines—AA, DL, and UA—operating between JFK and LAX airports from 2001 to 2023 is evident. Over this period, DL has experienced growth in market share, while AA and UA have faced a decline, indicating a correlation in passenger demand across these airlines.

**Figure 4:**
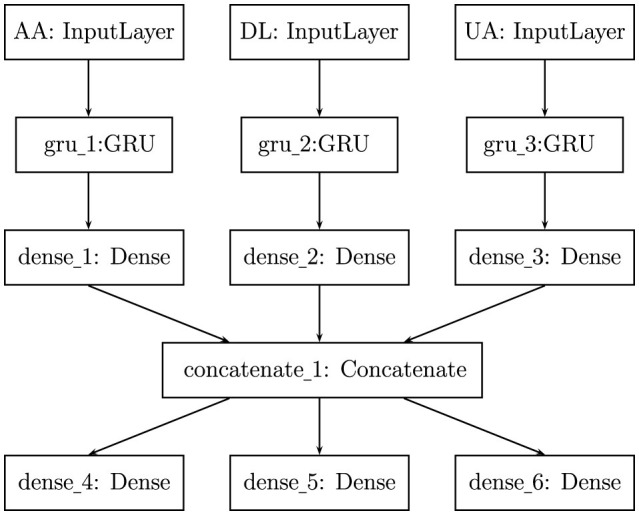
Architecture of our developed multiple input–output network to predict air passenger traffic for a route between two airports, JFK and LAX, for monthly number of passengers carried by American (AA), Delta (DL) and United (UA) during 2001-2019.

To run our proposed model on a test case of predicting air passenger traffic for individual routes between specific airport pairs, we first consider JFK (New York City) as origin airport and LAX (Los Angeles) as destination airport. For simplicity, we model the market using only three airlines, American (AA), Delta(DL), and United (UA), operating on the JFK to LAX route in a zero-sum game: If passenger demand for AA increases, then demand for other two airlines will drop (shown in Figure 3). We feed our multiple input-output network with three inputs, monthly number of passengers from JFK to LAX carried by American, Delta and United during 2001–2019. As depicted in Figure 4, each input time series is processed through a GRU layer with 50 neurons and a SoftPlus activation function, followed by a dense layer utilizing the ReLU activation function. The outputs from these layers are then concatenated and passed through three dense layers, each with 50 neurons and a ReLU activation function, to predict traffic for American, Delta, and United in 2020. The data preparation and hyperparameter tuning for this model follow the same approach as described for the single-input network above.

## 4 Conclusion

This study focused on predicting air passenger traffic and market share on specific routes between airport pairs, considering the competitive dynamics among various airlines operating concurrently. Recognizing that air traffic on these routes functions as a zero-sum game—where an increase in demand for one airline results in a decrease for others—we addressed the inherent interdependencies between competing airlines. To capture these complex relationships, we introduced a new variable representing the total number of passengers across all airlines on a given route, enabling a more comprehensive analysis of market dynamics.

To achieve accurate predictions, we developed a two-stage deep neural network algorithm for correlated time series forecasting. The first stage involved fitting four Recurrent Neural Network (RNN) models to generate univariate forecasts, with the Gated Recurrent Unit (GRU) model demonstrating the best performance in predicting aggregated market demand. In the second stage, the best-performing GRU model from Stage 1 was applied to each individual airline (disaggregated from the market), with all input tensors merged using the Concatenate function. This two-stage approach, developed using a multiple input and output neural network model, enhanced the model's predictive power by effectively leveraging the interdependencies among competing airlines.

All models were implemented in Python 3.6 (64-bit) using TensorFlow 1.8, with experiments conducted on a laptop equipped with an Intel i5-6200U CPU @ 2.30GHz and 16 GB RAM. To accommodate the complexity of handling multiple inputs and outputs, the Keras functional API was used instead of the Keras Sequential API. This methodology provides a nuanced understanding of passenger distribution across airlines, ultimately improving the ability to predict market dynamics and airline competition on specific routes.

## 5 Discussion

There are additional applications for this methodology. An application extension within the aviation industry of our proposed multiple input-output forecast model could be used to predict passenger demand for non-direct routings. Most U.S. airlines use the hub-and-spoke model, where flights go in and out of key hub cities where passengers change flights, for at least some share of flight operations. Aside from their usefulness for the analysis of U.S. commercial aviation passenger traffic, these models could have wider applications for the analysis of any industry in which market shares of competitors are correlated with one another. The ability to forecast demand is an attractive goal in and of itself and is highly valued in business and market research. The growth of computing power and the increased availability of advanced analytical software in recent years has dramatically augmented the capacity to actually execute demand forecasting. To this end, companies are making large investments in software, personnel, and consulting fees to carry out accurate demand forecasts.

Consider the problem of predicting market share among competitors in an industry in which any competitor's action affects the demand for all other competitors. That is, including market potential, if demand (or sales) for a competitor increases, then the demand for all other competitors decreases (other competitors lose their customers) and vice versa. In this study, we have examined the specific problem of forecasting the number of air passengers between two airports where several airlines operate on the route. Significant growth in the number of passengers flying one airline impacts the number of passengers flying on competitor airlines. Similar market structures exist in other industries, and our modeling may be suitable for broader application.

Additional examples of industries where this methodology could be applied include the automobile industry, where the launch of a new vehicle model by one manufacturer impacts the sales of competitors; the retail sector, where promotional campaigns or pricing strategies by one retailer can affect the sales volumes of others; and the telecommunications industry, where changes in pricing or plan offerings by one provider can influence customer churn and subscription rates across all competitors.

Other applicable contexts are the pharmaceutical industry, where new drug launches or patent expirations impact the sales of competing medications; the hospitality industry, where occupancy rates of one hotel chain are affected by the pricing and promotions of another; the banking and financial services sector, where the introduction of new products or changes in interest rates by one institution influences the customer base of others; and the streaming services market, where exclusive content releases and pricing adjustments by one platform can shift market shares among several competitors. Additionally, in the ride-sharing and transportation network companies (TNCs) sector, changes in fare structures, service availability, or new feature introductions by one company can directly influence demand for competing services.

In summary, these examples illustrate that our proposed multiple input-output forecast model has numerous applications beyond aviation. It can be effectively employed in any market where a few key players dominate, capacity is fixed in the short run, and customer choices for one provider typically exclude simultaneous purchases from another. Therefore, the methodology is broadly applicable across various industries where interdependent competition exists, providing valuable insights into market share dynamics under different competitive scenarios. The relevant parameters need to be investigated further, but we can conclude early on that there are numerous industries and situations for which this methodology may be applied.

## Data Availability

The original contributions presented in the study are included in the article/supplementary material, further inquiries can be directed to the corresponding author.
